# Clinical Study of Effects of Jian Ji Ning, a Chinese Herbal Medicine Compound Preparation, in Treating Patients with Myasthenia Gravis via the Regulation of Differential MicroRNAs Expression in Serum

**DOI:** 10.1155/2014/518942

**Published:** 2014-01-05

**Authors:** Chao Jiang, Ping Liu, Jingsheng Zhang, Wenjing Bao, Shaobo Qiu, Yan Liang, Lin Jiang

**Affiliations:** ^1^Longhua Hospital Affiliated to Shanghai University of Traditional Chinese Medicine, No. 725, Wanping Nanlu, Xuhui District, Shanghai 200030, China; ^2^The China Affiliated Hospital of Liaoning University of Traditional Chinese Medicine, Shenyang City, Liaoning 110032, China; ^3^State Key Laboratory of Bioreactor Engineering & Shanghai Key Laboratory of New Drug Design, School of Pharmacy, East China University of Science and Technology, Shanghai 200237, China

## Abstract

Myasthenia gravis (MG) is an autoimmune disease, of which the pathogenesis has remained unclear. At present, MG does not have any effective treatment with minor side effects. Jian Ji Ning (JJN), a traditional Chinese medicine formula consisting of 11 medicinal plants, has been used in the treatment of MG for many years. The present study aims to determine if the Chinese herbal medicine JJN could lighten the clinical symptoms of patients with MG via the regulation of differential microRNAs (miRNAs) expression in serum. JJN should be orally administered twice a day for 6 months. In the efficacy evaluation adopting the Quantitative Myasthenia Gravis Score (QMG), we found that JJN could improve the clinical symptoms of patients with MG more effectively. Besides, we found that JJN could regulate differential miRNAs expression in serum of patients with MG. Accordingly, we speculate that the effects of JJN on improving clinical symptoms and blood test indicators of patients with MG may be due to its inhibition of apoptotic pathways of some immune cells and its connection with the regulation of serum miRNAs of some patients. In conclusion, we believe that JJN has a reliable curative effect on patients with MG-induced neuropathologic changes.

## 1. Introduction 

Myasthenia gravis (MG) is an antibody-mediated neuromuscular transmission chronic disorder, with an incidence rate of 3–30/1,000,000 people per year. The targets are postsynaptic proteins, mainly involving the skeletal muscle acetylcholine receptor (AchR) and the muscle-specific tyrosine kinase (MuSK) [1, 2]. Some studies have shown that genetic factors played an important role in pathogenesis of MG [3]. In addition, infection with viruses or bacteria, such as poliovirus and *Escherichia coli*, may be involved in the pathogenesis of MG [4, 5]. However, no report has clearly elucidated the fundamental pathogenesis of MG but merely some accepted hypotheses. The main clinical characteristic of this disease is the volatility of skeletal muscle weakness. At present, anticholinesterase drugs, nonspecific immunosuppressants, thymectomy, and plasmapheresis are main treatments for MG [6–8]. Unfortunately, the abovementioned treatments have some serious side effects, such as cardiac arrhythmia, osteoporosis, and hypotension and cannot inhibit the relapse of symptoms of patients with MG and/or achieve complete remission [9]. Therefore, alternative treatments with higher efficacy and fewer side effects are required. Traditional Chinese medicine (TCM) has been practised for many diseases, including cancer, cardiovascular disease, inflammation, and MG's disease, owing to its long-term efficacy and few side effects [10–14].

Jian Ji Ning (abbreviated as JJN), a traditional Chinese herbal medicine, consists of eleven herbal components, including *Hedysarum multijugum* Maxim. 50 g, *Pseudostellaria heterophylla* (Miq.) Paxex Pax et Hoffm. 25 g, *Atractylodes macrocephala* Koidz. 15 g, *Citrus aurantium* L. 15 g, *Cimicifuga heracleifolia* Kom. 10 g, *Leonurus japonicus* Houtt. 30 g, *Saposhnikovia divaricata* (Turcz.) Schischk. 10g, *Angelica sinensis* (Oliv.) Diels 10 g, *Lycium barbarum* L. 15 g, *Polygonum multiflorum* Thunb 15 g, and *Cornus officinalis* Sieb. 15 g. JJN has been mainly used to treat the autoimmune disease myasthenia gravis for many years. Our previous studies show that JJN is effective in various experiments and clinical treatments related to MG. It can reduce the level of IFN-1, regulate mRNA expression, reduce AChRAb level in blood, improve the severity of myasthenia gravis, promote the growth of new axon in neuromuscular junction (NMJ), increase the number of synaptic vesicles and reduce the synaptic injury in model rat of trial autoimmune MG (EAMG), and effectively regulate the expression levels of some EAMG-related differential proteins [15–22]. However, our understanding of the treatment of MG patients by JJN is still at the molecular level, so that the influence is limited.

MicroRNAs (miRNAs) are small (∼22 nucleotides long), noncoding RNAs that mediate posttranscriptional silencing of target genes. In animals, miRNAs usually bind to complementary sites in the 3′ untranslated region (UTR) of target genes and regulate target gene expression by either translational inhibition or miRNA degradation or both [23]. Ever since the discovery of miRNAs, researchers have been committed to determining biological functions of miRNAs and their related diseases. Dysregulation of miRNAs has been associated with certain human diseases, such as leukemia and heart disease [24, 25]. Moreover, to the best of our knowledge, no report has shown that there are changes in the pattern of differential microRNA expression in serum of MG patients who have received JJN treatment.

According to the above discussion, our study aims at determining whether Chinese herbal medicine JJN could reduce muscle weakness and neuronal apoptosis of autoimmune-induced MG disease via regulation of differential microRNAs expression in serum of patients with MG.

## 2. Materials and Methods

### 2.1. Participants

This study was approved by Ethics Committee of the Longhua Hospital Affiliated to Shanghai University of TCM, and all participants were provided with informed consents on this study according to the rules of the Ethics Committee. All of them were Chinese Hans. There were 60 samples in total, including 35 males and 25 females at an average age of 48.70 ± 16.45 years and with a disease duration of 28.03 ± 23.83 month (mean ± SD). All of them were patients who were diagnosed with MG in the Longhua Hospital Affiliated to Shanghai University of TCM and the China Affiliated Hospital of Liaoning University of TCM from November 2010 to April 2012. According to the Osseman classification [26], all MG patients were included in this study because they met the inclusion criteria but not the exclusion criteria. All patients included in this study belonged to class II (33 class IIa; 27 class IIb). Moreover, the study collected peripheral blood samples from 10 healthy individuals as the control group, including 3 males and 7 females at an average age of 45 ± 18 years.

The diagnosis of MG was based on the diagnostic criteria for autoimmune myasthenia gravis in western medicine and the medical diagnosis of flaccidity in TCM [27, 28]. The inclusion criteria are shown as follows: (1) patients with MG belonging to the modified Osseman I, IIA, IIB type; (2) patients diagnosed with spleen-kidney deficiency type flaccidity in TCM, who have one or more cardinal symptoms and secondary symptoms, including pulse and tongue conditions; (3) patients aged from 14 to 75 years; (4) patients informed and willing to participate in this study. The patients with familial MG, congenital myasthenia syndrome (D-penicillamine, interferon induced MG, etc.), allergy (allergic to 2 or more kinds of food or drugs), and serious complications, such as cerebral vascular diseases, renal insufficiency, hematopoietic system diseases, and mental illness, the patients who participated in clinical trials of other drugs in the past month, and the patients who received the treatment with plasma exchange or intravenous gamma globulin in the past three months will be excluded. Ten gender-matched healthy donors with no history of autoimmune disease will be included in this study as the control group. The concomitant use of medicines other than the TCM used in this study is not allowed. After assessment by a neurologist, eligible patients will be assessed by a TCM practitioner to ensure their eligibility.

### 2.2. Herbal Preparation

In this study, we used Chinese herbal decoctions prepared according to the formula of TCM of Professor Jingsheng Zhang at Affiliated Hospital of Liaoning University of TCM. This formula specific for the TCM syndrome matching certain MG symptoms has been proved to be effective in the early clinical and experimental studies. JJN was supplied by the GMP plant of Benxi Chinese Medicine Factory (Liaoning Province, China). The preparation was a mixture of 11 Chinese herbal medicines as described above. In brief, the drugs were extracted with standard methods according to Chinese Pharmacopoeia (China Pharmacopoeia and Committee, 2000). These crude drugs were soaked in distilled water and boiled for 30 min twice. After that, the drug solution was filtered through a mesh, then concentrated to 4 gmL^−1^ by a vacuum pump at room temperature, and finally stored at −20°C till being used. All herbs were obtained from qualified suppliers in Liaoning Province, China and authenticated at the Chinese Manufacturers Association Testing and Certification Laboratories and the Research and Analysis Laboratory on the basis of standards specified in the Pharmacopoeia of China (2005 edition). These tests included macroscopic and microscopic examination of cross-sections and powders, chemical tests, and/or chromatographic analyses. The screening of heavy metals and pesticides and a microbial limit test were required to ensure the safety of participants in use. All medicine packages were distributed by an independent researcher in another room after the patients' visits. Then, patients were instructed to dissolve the granules in each package in hot water and drink it twice a day (the daily dose for adults is 250 mL and was administered orally two times a day before each meal). Changes were not allowed in the studied herbal medications during the study.

### 2.3. Study Design and Therapeutic Method

**Figure 1:**
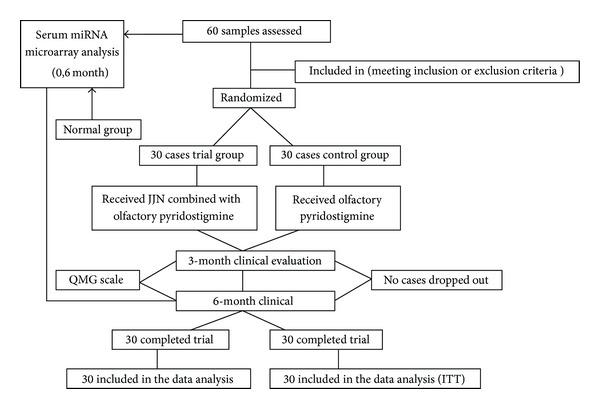
Clinical trial profile.

This is a prospective and randomized controlled study. Random numbers generated by random-list generator software were assigned to the participants. For more confidentiality, we used patients' codes at all follow-up phases. All subjects had been given written information and a verbal explanation concerning the study before they agreed to participate in the study. After one-week baseline assessment, the subjects were randomly assigned into either the treatment combining JJN (usage as above described) with pyridostigmine bromide (60 mg, 4 times a day, manufactured by Sunway Pharmaceutical Co., Ltd., Shanghai, China, approval no. H31020867) or the treatment with pyridostigmine bromide (using treatment allocation codes generated by a statistician and designed to ensure balance of gender, age, and severity of MG between groups (Figure 1)). All the patients were treated with JJN orally twice per day for 6 months, and the dose of JJN remained unchanged. However, the dosage of pyridostigmine bromide can gradually decline to a relatively low maintenance level according to the improvement condition of symptoms of patients. See some literatures for specific methods [29]. The study was started in November, 2010, and finished in April, 2012.

### 2.4. Preparation of miRNA Microarray

First, put the 5 mL blood sample collected from each subject into an EDTA-supplemented tube and mix it with 3 mL PBS, and then put 6 mL Ficoll-Paque mixture (GE Biosciences, Pittsburgh, PA, USA) into the tube and centrifuge the tube at 2000x rpm for 20 min. The separation method of peripheral blood mononuclear cells is as shown in the literature [30]. The cells are applied to RNA extraction. Total RNA is extracted from lymphocyte cells according to the instructions of the manufacturer of Mirvana miRNA Isolation Kit (Ambion, Carlsbad, USA). Next, we submit the samples collected from 3 MG patients (pretreatment), 2 MG patients (treatment), and 3 controls to Shanghai Biotechnology Corporation for hybridization of Agilent Human miRNA array (v.12.0). Each microarray chip is hybridized with a single sample labeled with either Cy3 or Cy5. The hybridization should follow background subtraction and relevant specifications. The quality control standard of miRNA microarray is 2100 RIN> = 6.0 and 28S/18S> = 0.7. Only when the miRNA microarray is up to the standard, we can move into the next phase.

### 2.5. miRNA Microarray Data Analysis

#### 2.5.1. MultiClassDif (Multiclassification with Differences) Analysis

We transformed all raw data derived from the miRNA microarray into log_2_ with zero mean and unit sample variance and normalized expressions and analyzed them. In order to evaluate the effect of JJN in treating MG patients more accurately, we compared the miRNAs expressions of normal people, MG patients (pretreatment), and MG patients (treatment), respectively. Log ration of 0, a median over all patients that made each patient with a normalized expression level, was used to find the further normalization of relevant miRNA expression levels. The random variance model (RVM) *t*-test was used to calculate the weighted differential expressions of miRNAs between the MG (before and after treatment) and the normal. The differential expressions of miRNAs with the fold-change >1.5 and *P* < 0.05 were considered significant. The analysis aimed at finding out the differential expressions of miRNAs for regulation of all target genes through searching the mirdb database. The heat map analysis and hierarchical cluster analysis of expression data adopted Cluster 3.0 and TreeView programs. The analysis method was MultiClassDif [31–33].

#### 2.5.2. Analysis of the Significant Trend of miRNA Expressions

We selected differential expression genes on the basis of the logical sequence that corrected analysis of variance according to RVM. In accordance with the variation trend that a gene had different signal density under different situations, we identified a unique model expression tendency. The value of the original expression was converted into log_2_ ratio. By using the strategy of clustering short time-series gene expression data, we defined some unique profiles. The expression model profiles are related to the actual or the expected number of genes assigned to each model profile. Significant profiles have higher probability than that expected by Fisher's exact test and multiple comparison test [34–36]. We carried out significance analysis of gene expression trend by means of Series Test of Cluster (STC) analysis based on the screening as previously described.

#### 2.5.3. Gene Ontology (GO) Analysis

GO analysis was applied in order to organize genes into hierarchical categories and uncover the miR-Gene regulatory network on the basis of biological process and molecular function [37]. In detail, two-side Fisher's exact test and *χ* ^2^ test were often used to classify the GO category, and the false discovery rate (FDR) [38] was calculated to correct the *P* value. We chose only GOs that had a *P* value of <0.001 and an FDR of <0.05. Within the significant category, the enrichment Re was given by

(1) $$\begin{array}{c} Re=\frac{n_{f}/n}{N_{f}/N}, \end{array}$$

where *n*_*f*_: indicates the number of differential genes within the particular category, *N*_*f*_: indicates the number of differential genes in the entire microarray, *n*: indicates the total number of genes within the same category, and *N*: indicates the total number of genes in the microarray.

Afterwards, the metastasis-related network of miRNA-mRNA interaction, representing the critical miRNAs and their targets, was established according to the miRNA degree.

#### 2.5.4. Enrichment Analysis of Target Genes

The significance analysis of inferred miRNA target genes cannot be performed until we demonstrate the significant (*P* < 0.05) difference among the normal, MG (pretreatment), and MG (treatment) samples in the expression of miRNA, accomplish the pathways analysis [39, 40] of these genes on the basis of DAVID online analysis system, and correct *P* value by FDR. The genome containing less than 5 genes overlapping will be removed from the DAVID analysis. In our analysis, GO terms and pathways with an FDR-adjusted *P* value of <0.05 will be retained.

#### 2.5.5. To Construct MicroRNA-GO-Network

The miRNA-GO-network was built according to the relationship between significant GOs and genes and the relationship between miRNA expressions and gene expression [41]. The adjacency matrix of miRNA and genes *A* = [*ai*, *j*] was determined by the attribute relationship between GOs and miRNA, and *ai*, *j* represented the relation weight of GO *i* and miRNA *j*. In the miRNA-GO-network, the circle represented gene, the square represented miRNA, and the edge represented the relationship between gene and miRNA. The center of the network was represented by degree. The degree represented the relationship between one miRNA or GO and the GOs or miRNAs around. The key miRNA and GO in the network had the highest degree.

### 2.6. Assessment of Clinical Effects

Baseline evaluation was performed before randomization. The 13 Quantitative Myasthenia Gravis scores (QMG-13 including all dimensions and the single index) [42] were often used to monitor the quality of life, the extent of MG and muscle weakness of patients according to the assessment of MG related clinical symptoms. The follow-up visits to the clinical assessment were held for all patients within 3 or 6 months' treatment. The blood collection for serological data was conducted only at the first and the last visit (6 months). The serological data included RNA extraction and renal and liver functions tests. The reported adverse events (AEs) were observed by the patient or the investigator and recorded spontaneously. The severity, outcome, and hypothetical cause of each AE report were assessed and recorded.

### 2.7. Statistical Analysis

Data were expressed as mean ± SE. It was of statistical significance to use Wilcoxon signed rank test to assess the difference before and after JJN treatment. The difference between the two groups was assessed by means of Mann-Whitney *U* test. The screening for differential miRNA expression adopted RVM *t*-test while GO-analysis and Pathway analysis adopted Fisher's exact test and *χ* ^2^ test. A *P*-value <0.05 was regarded as significant. The SPSS version 14.0 (SPSS Inc., Chicago, IL) was used for the statistical computation.

## 3. Results

### 3.1. Bioinformatics Analysis

#### 3.1.1. Differentially Expressed miRNAs of MG and Normal

**Figure 2:**
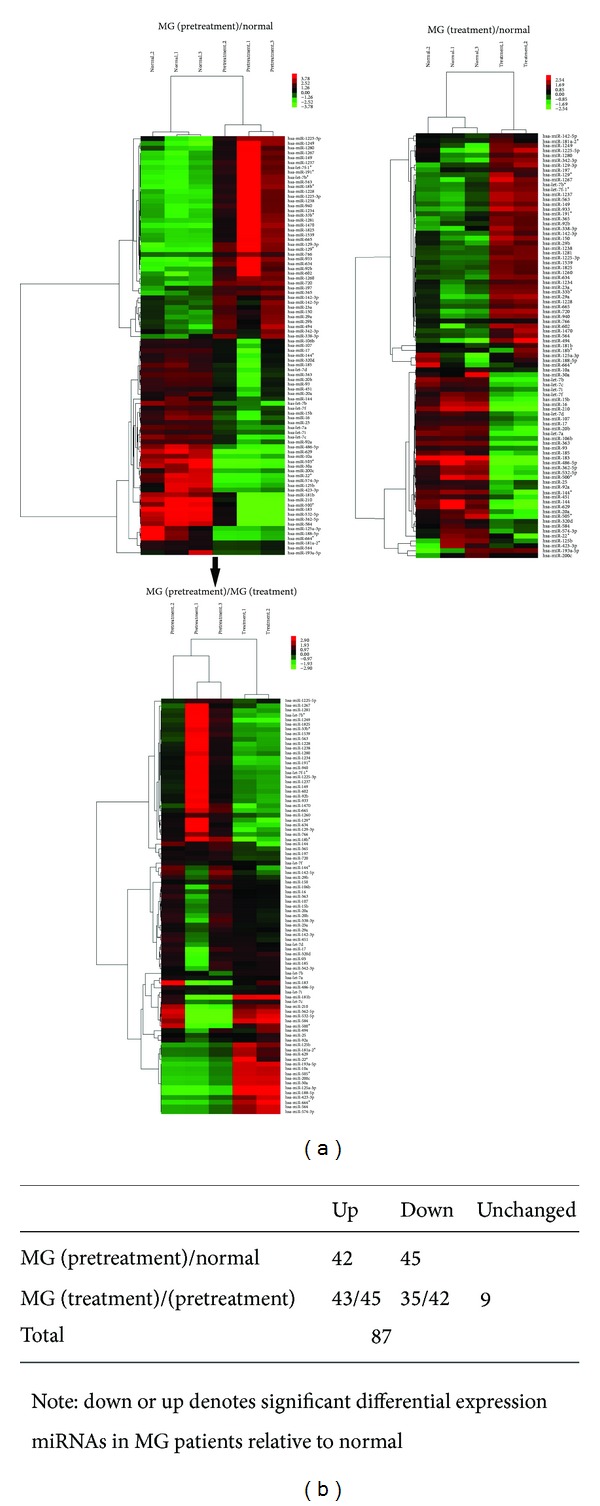
(a) Heat map of differentially expressed miRNAs between the MG (pretreatment)/normal and MG (treatment)/normal is shown at the horizontal, and the heat map of differentially expressed miRNAs between the MG (pretreatmentand) the MG (treatment) is shown at the vertical. (b) Significant differentially expressed miRNAs of three groups.

The multiple comparison test based on *P* value <0.01 and FDR <0.05 revealed a total of 87 significant differential gene expressions in three different genetic screening groups consisting of MG (pretreatment), MG (treatment), and normal samples. Hierarchical cluster analysis revealed that the differential expression profiles of miRNAs extracted from these samples were roughly classified, respectively. The consecutive heat maps were shown in Figure 2(a) and the significant differential expressions of miRNAs were shown in Figure 2(b). On the heat maps, we noted that there were big changes in the miRNAs expression profiles of MG (pretreatment)/normal, MG (treatment)/normal, and MG (before and after treatment), which indicated that effects of TCM compound preparations JJN in treating MG patients may be realized via regulation of miRNA expression profilles.

#### 3.1.2. STC Analysis

**Figure 3:**
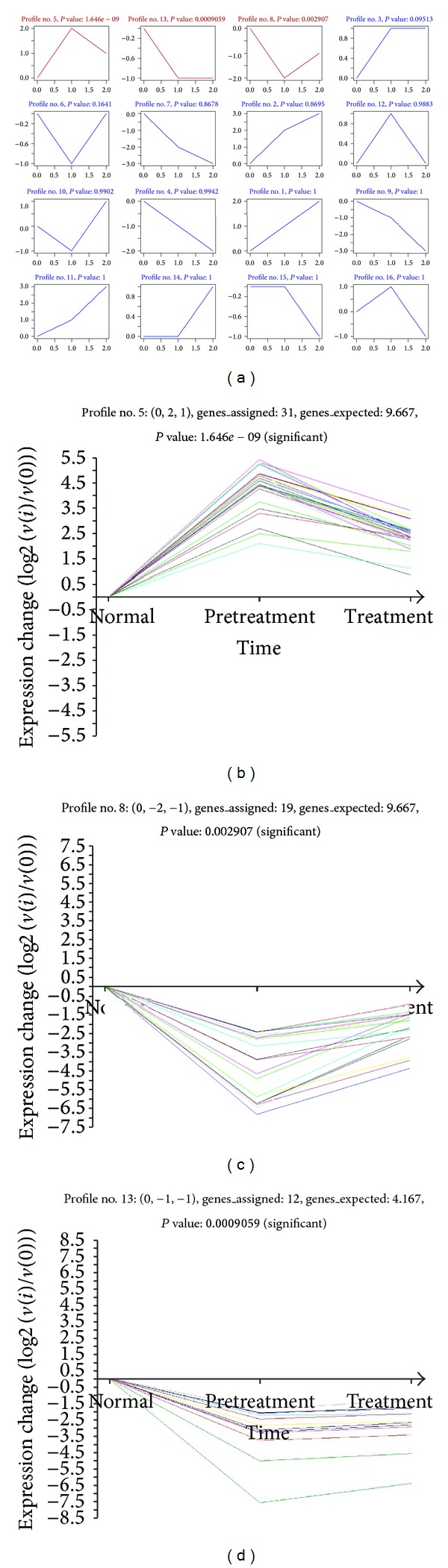
Differences in gene expression trends. Expression of each box represents a trend, a trend marked as each box number, such as 5, 8, and 13, lower marked with *P* value, that is, the trend level of significance, on behalf of the trend test for multiple comparisons after a significant correction level (*P* value), color-coded for significant trends, such as the trend of 5, 8, and 13. Significant trends of the selection criteria: *P* value∖0.05/16 = 0.0031. As shown above, miRNA expression of differences in a total of 16 species of trends, including the three trends is the expression of significant trends (*P*∖0.0031), which are plotted over the color part of the trend. (Color figure online.)

Next, we analyzed miRNA expressions in MG (pretreatment), MG (treatment), and normal samples and observed the significant trend of miRNA expression (Figure 3). Every trend stands for a miRNA group with the similar expression trend. The horizontal axis represents duration or different courses and the vertical axis represents the logarithm of ratio of the control signal value of a miRNA expression. We listed three different sample lines representing three different courses (normal, pretreatment, and treatment). *P* value represents the significance level of the actual number of randomly distributed miRNAs. When *P* value is smaller, the impact of the analysis of the trend of miRNA expression will be more remarkable.

We worked out 16 differential miRNA expression trends by using the significant expressions of gene arrays, including three significant trends (*P*∖0.0031; 0.05/16 = 0.0031), which were plotted over the color part (up left of Figure 3, zoomed on the low). The significant trend was like Profile no. 5 and the trend change followed (0, 1, 2, 2). ‘‘One” or ‘‘2” did not represent the actual expression value but symbolized the classification of miRNA expression level. The no. 5 significant trend showed that the level of miRNA expression was drastically upward from normal to pretreatment, but during the course from pretreatment to treatment, the level of miRNA expression appeared downward. From the no. 13 significant trend, we could find that, during the course from normal to pretreatment, miRNA expression was drastically declining, and then the miRNA expression declined to the minimum level and remained unchanged. From the no. 8 significant trend, we could find that, during the course from normal to pretreatment, the miRNA expression was also declining sharply, but after entering the aftertreatment stage, the miRNA expression appeared upward and this trend lasted until the treatment stage. Finally, we selected the most simple no. 5 trend as the best significant trend of this study. After crosstab of target genes of miRNAs and multiClassDif analysis of differential gene expressions of miRNAs, we obtained genes and corresponding miRNAs (data not shown).

#### 3.1.3. GO Analysis and Pathway Analysis

**Figure 4:**
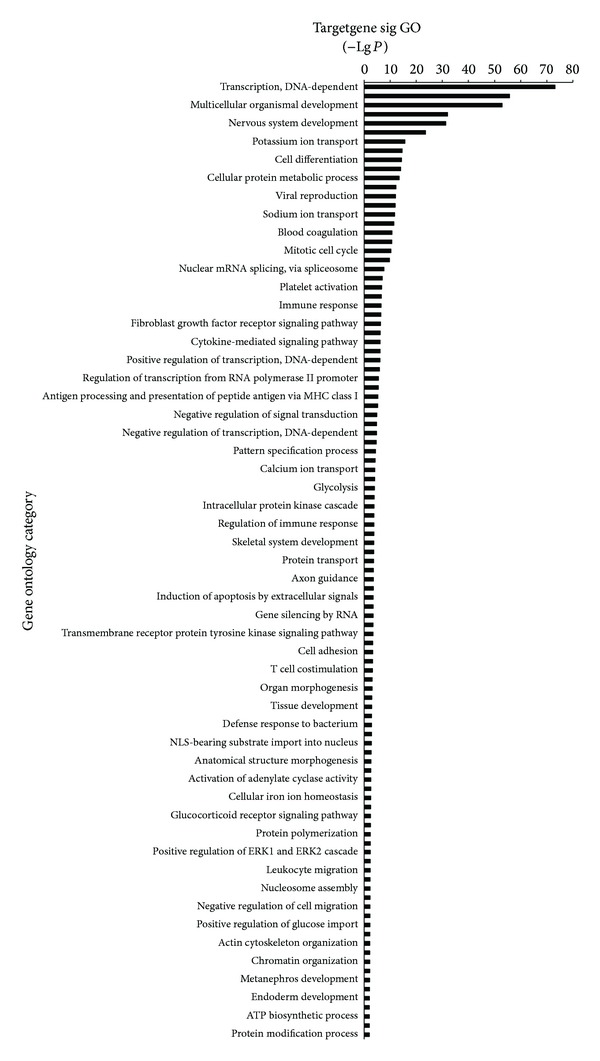
The significant GO functional maps of the target genes. Ordinate is the name of the target gene function and the abscissa is the *P* value of the negative logarithm. A higher number indicates that the function is more significant.

**Figure 5:**
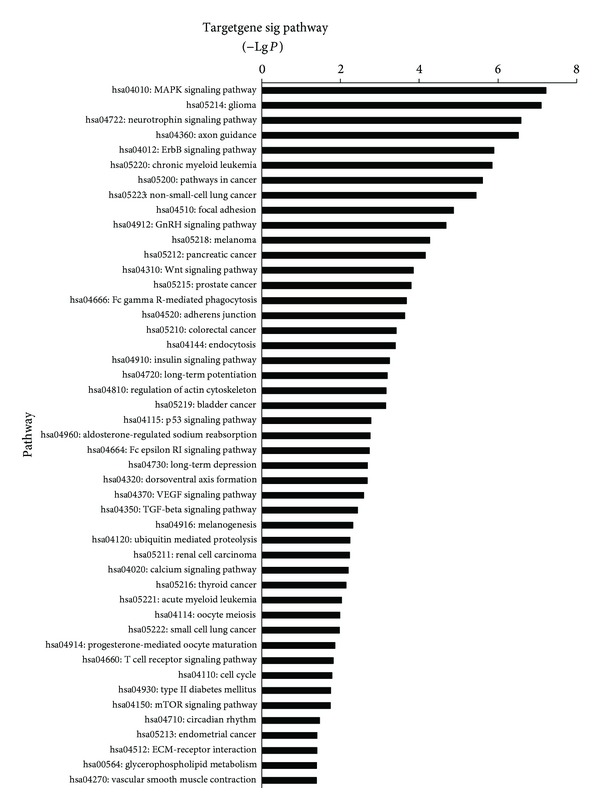
KEGG pathway associations of the target genes of the miRNAs. The enrichment scores of biological processes are shown as –log(*P* value).

Next, we performed GO analysis to organize the differentially expressed genes and miRNAs on the basis of biological process and function. The results were shown in Figure 4. We found that, compared with the normal group, the differential miRNAs of the MG group (pretreatment) was upward or downward while the MG group (treatment) was upward or downward. The corresponding target genes involved 105 significant differential miRNAs. The points of significance include (−Lg*P* ≥ 20) transcription, DNA-dependent, ion transport, multicellular organismal development, chromatin modification, nervous system development, and gene expression. Furthermore, we did pathway analysis to find out significant pathways of relevant differentially expressed genes or miRNAs. As described in Figure 5, we found that target genes involved 47 significant pathways in total, including (−Lg *P* ≥ 5) MAPK signaling pathway, glioma, neurotrophin signaling pathway, axon guidance, ErbB signaling pathway and chronic myeloid leukemia, pathways in cancer, and non-small-cell lung cancer.

#### 3.1.4. MicroRNA-GO-Network

**Figure 6:**
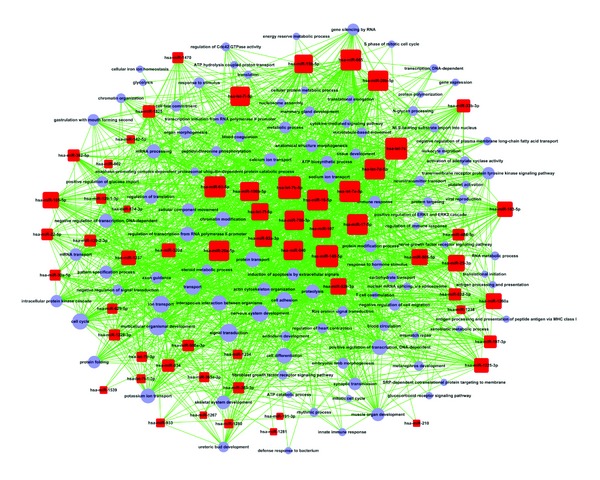
MicroRNA-GO-network. Red rounded rectangle nodes represent microRNA, and light purple cycle nodes represent GO. Lines of edge show the regulatory relations at microRNA and GO. Color and shape indicated no special significance but better distinguish between network MicroRNA and GO. The more MicroRNA regulates GO, the more the area of MicroRNA occupies, similarly, the more GO could be regulated by MicroRNA, the more the area is also occupies, Similarly, the more GO by MicroRNA regulated, the greater its area is also. Its quantitative relationship can be provided by the corresponding tables.

To further explore the relationship between miRNAs and gene function, we built the miRNA-GO-network among the three groups (Figure 6). As shown in Table 1, the 17 key miRNAs (degree ≥ 50) in the network were hsa-let-7b-5p, hsa-miR-149-5p, hsa-let-7c, hsa-miR-93-5p, hsa-let-7a-5p, and so on. In the miRNA-GO-network, the degree represented the relationship between a miRNA or GOs and the GOs or miRNAs around. The three key miRNAs (degree ≥ 70) in the network, that is, hsa-let-7b-5p, hsa-miR-149-5p, and hsa-let-7c, had a high degree. As we can see from Table 2, the 22 key GOs (degree ≥ 30) in the network were signal transduction, multicellular organismal development, ion transport, and signal transduction in the GO among which multicellular organismal development and ion transport in the GO were more regulated by microRNA. This showed the differences of different samples of MG patients before and after treatment using JJN. Among them, hsa-let-7b-5p had the highest degree in the network. Therefore, hsa-let-7b-5p was selected for further study.

### 3.2. Comparison of the QMG Scoring between the Two Groups before and after Treatment

The result of baseline evaluation was shown in Table 3 and demonstrated the comparison condition between the two groups before randomization. The QMG scores of the trial group and the control group were decreased after treatment. There was a significant difference between the two groups (*P* < 0.05). However, after 3 and 6 months' treatment, the QMG scores of the trial group were apparently lower than those of the control group (*P* < 0.05); moreover, there was also an obvious difference between the two groups compared with the same group before treatment (*P* < 0.05, Table 4).

## 4. Discussion

As for many autoimmune diseases, it is known that the triggering events involved in MG are not clearly defined. Complex disease pathogenesis of MG has hindered the advancement of our understanding of disease initiation, thus delaying the identification and treatment of susceptible individuals. Obviously, it is necessary to investigate the pathogenesis of MG more deeply and seek more rational and effective treatment for MG, which no doubt becomes a hot spot in the field.

According to the previous studies, miRNAs were small nucleotides of RNA that participate in the regulation of a variety of cellular processes, including cell differentiation, cell cycle progression, and apoptosis. Ever since the discovery of miRNAs, tremendous effort has been devoted to determining the biological functions of miRNAs and their relevance to diseases. Moreover, miRNAs emerge as important factors in tumorigenesis and metastasis and their expression signatures are associated with the prognosis and progression in chronic lymphocyte leukemia and lung cancer [43, 44]. In addition, bioinformatics analysis reveals that miRNAs can control the expression of one-third of the human proteome [45]. Recent evidence showing miRNA as a micromanager of various stages of immune regulations has generated interest in the involvement of miRNAs in autoimmune disorders. Although still at an early stage in understanding their impact on immunity, miRNAs are changing the way we think about the development of the immune system and regulation of immune functions [46]. In addition to our preliminary previous study, it seems that no report on the biological consequences of miRNA dysregulation in MG has been characterized, particularly in relation to therapeutic intervention by taking Chinese herbal compound.

TCM is a medical system, and discoveries of ancient Chinese evolved through at least 3000 years of uninterrupted clinical practice. The TCM treatment usually requires a traditional diagnosis method to distinguish the TCM syndrome, which is based on clinical symptoms and signs followed by the use of individualized treatment [47]. What is more, Chinese herbal medicines have been used for thousands of years and are beneficial in prevention and treatment of many diseases, including MG. Greater attention is being given to such medicines due to their varied biological actions and low toxicity. According to our previous studies, JJN, a formula designed according to the TCM theories and clinical experience, has been used to treat MGI (I and II type) patients in China for decades. However, this study is the first report to address the therapeutic effects of JJN on patients induced serum microRNAs expression differences in MG.

In this study, array-based gene and miRNA expression profiling were performed on three sample groups, that is, MG sample group (pretreatment), MG group (treatment) and normal group. First, the findings support the notion that there are 87 significant differentially expressed miRNAs in the three groups. It means that the MG group (pretreatment), MG group (treatment), and normal group are completely different in the miRNA expression level. Among them, the miRNA expression change in MG group before and after treatment may have occurred due to therapeutic effects of Chinese herbal compound JJN on MG patients. Next, we observed significant trend of miRNA expression in three groups at three different courses (normal, pretreatment, and treatment) and found that the upward or downward trend of MG patients was corrected compared to the normal group, after treatment of Chinese herbal compound JJN. It means that JJN has some therapeutic effects on patients with MG as the prolongation of treatment time. Then, we performed GO analysis and found that there were certain significant differences in miRNAs features between MG group (before and after treatment) and normal group and the corresponding target genes involved those above significant features. It is tempting to speculate that Chinese herbal compound JJN was applied to the treatment of MG because it had a certain impact on the expression of above significant differential miRNAs features of the human beings and JJN may play a therapeutic role via the regulation of miRNAs expression profile changes in these functions. Furthermore, we applied DAVID analysis to calculate each target gene and conduct pathway analysis, accordingly finding out that target genes involved 47 significant pathways in total. Then, we conjectured that the effect of Chinese herbal compound JJN in treating MG came into play in the abovementioned way. In the biological network, as we all know, one advantage is that the network contains interaction information, which provides an intuitive way to explore gene functions in context using visualization approach. Such holistic approach has advantages for gene expression modules during both disease and its development for their regulation [48], and expression levels are the highest correlation across samples [49]. As a class of gene regulators, combinatorial regulation is an important feature for miRNA. Usually, a given miRNA may have multiple different mRNA targets, whereas a given target gene may also be targeted by multiple miRNAs [50]. In this study, the integrated bioinformatics analysis from gene and miRNA expression profiling suggested that 17 miRNAs, that is, hsa-let-7b-5p, hsa-miR-149-5p, hsa-let-7c, hsa-miR-93-5p, hsa-let-7a-5p, hsa-miR-665, hsa-miR-16-5p, hsa-miR-17-5p, hsa-miR-20b-5p, hsa-miR-20a-5p, hsa-let-7d-5p, hsa-miR-940, hsa-miR-766-3p, hsa-let-7i-5p, hsa-miR-106b-5p, hsa-miR-15b-5p, and hsa-miR-107, were associated with the effect of JJN in treating patients with MG. Among them, hsa-let-7b-5p showed the highest degree in miRNA-GO-network, which was built to explore the association of miRNAs with gene or gene ontology. From our previous studies, we know that key regulatory mechanism by dysregulation of let-7 family was involved in MG, which inhibited IL-10 expression in Jurkat cells. All the results suggest a possible link between the miRNA-mediated mechanisms and the pathogenesis of MG. The reason why the symptoms of MG are partially reversed may be the increase in the level of let-7c in MG patients [25]. Moreover, previous studies have reported that let-7 microRNAs are principal regulators that control major cell functions in various physiological and pathological processes [51]. Collectively, the above-described evidence also suggests that Chinese herbal compound JJN may play a therapeutic role in MG by regulating the let-7 level.

Based on the abovementioned microarray bioinformatics analysis, we obtained that Chinese herbal compound JJN plays its therapeutic effect in treating MG patients by regulating differences in miRNA expression profiles, differences in the certain functions of miRNA expression profiles, changes in miRNA expression pathways, and other ways. Then, from a macroperspective, it is necessary to evaluate the clinical efficacy to further validate therapeutic effect of JJN on MG patients. In this study, on the basis of western conventional treatment, we pulsed Chinese herbal compound JJN to treat MG and found that the QMG score of the trial group was apparently lower than that of the control group before and after treatment; there was a significant difference between the two groups (*P* < 0.05), with statistical significance. MG patients were able to significantly reduce the simple western medicine usage and the clinical symptoms can be effectively certainly alleviated without side effects. In addition, larger samples of previous clinical studies have also proven that the clinical obvious effective rate and effective rate of JJN therapy in MG patients were 71.62% and 87.84%, respectively [19].

In conclusion, through analyzing miRNA microarray data in MG, we provided evidence for the proposition that there were great differences among the MG group (pretreatment), MG group (treatment), and normal group on the basis of MultiClassDif, STC analysis, GO analysis, Pathway analysis, and microRNA-GO-network and indicated that the effects of Chinese herbal compound JJN on treating MG patients might be realized via the regulation of changes in expression profiles of those miRNAs. Meanwhile, Chinese herbal compound JJN provides more clinical advantages than corticosteroids for MG treatments.

## Figures and Tables

**Table 1:** The key microRNA in network (degree ≥ 50).

MicroRNA	Degree
hsa-let-7b-5p	73
hsa-miR-149-5p	71
hsa-let-7c	70
hsa-miR-93-5p	66
hsa-let-7a-5p	66
hsa-miR-665	65
hsa-miR-16-5p	64
hsa-miR-17-5p	64
hsa-miR-20b-5p	62
hsa-miR-20a-5p	62
hsa-let-7d-5p	62
hsa-miR-940	58
hsa-miR-766-3p	58
hsa-let-7i-5p	58
hsa-miR-106b-5p	56
hsa-miR-15b-5p	54
hsa-miR-107	52

**Table 2:** The key GO in network (degree ≥ 30).

GO_name	Degree
Signal transduction	52
Multicellular organismal development	52
Ion transport	48
Nervous system development	47
Transport	44
Cell adhesion	44
Cell cycle	43
Cell differentiation	41
Positive regulation of transcription, DNA-Dependent	40
Interspecies interaction between organisms	40
Protein transport	39
Chromatin modification	38
Potassium ion transport	37
Regulation of transcription from RNA Polymerase II promoter	36
MRNA processing	35
Proteolysis	34
Regulation of translation	34
Skeletal system development	33
Negative regulation of transcription, DNA-dependent	33
Calcium ion transport	31
Muscle organ development	30
Actin cytoskeleton organization	30

**Table 3:** Comparison of baseline evaluation between the two groups $\left(\overset{-}{x}\pm s\right)$.

Group	Case	Gender	Age	Disease course
		(male/female)	(year, range)	(month)
Normal	10	3/7	45 ± 18	
Trial group	30	12/18*	48.70 ± 16.45 (17–75)**	28.03 ± 23.83^△^
Control group	30	13/17	49.03 ± 15.26 (22–75)	30.43 ± 29.69

Notes: *P > 0.05, compared with the control group (gender); **P > 0.05 (age), ^△^ *P* > 0.05, compared with the control group (disease course).

**Table 4:** Comparison of evaluation of QMG scoring among the two groups $\left(\overset{-}{x}\pm s\right)$.

Item	Trial group (*n* = 30)	Control group (*n* = 30)
Pretreatment	10.80 ± 4.92*	12.10 ± 5.70
3 months	7.90 ± 3.82^∗∗▲^	10.86 ± 4.98
6 months	6.19 ± 3.57^△▲^	9.41 ± 5.18

Notes: *P > 0.05, compared with the control group (Pretreatment); **P < 0.05 (3 months), ^△^ *P* < 0.01 (6 months), compared with the control group; ^▲^ *P* < 0.05 (3 months and 6 months) compared with the same group before treatment.
